# A new biomarker combining multimodal MRI radiomics and clinical indicators for differentiating inverted papilloma from nasal polyp invaded the olfactory nerve possibly

**DOI:** 10.3389/fneur.2023.1151455

**Published:** 2023-03-21

**Authors:** Lianze Du, Qinghai Yuan, Qinghe Han

**Affiliations:** Radiology Department, The Second Hospital of Jilin University, Changchun, China

**Keywords:** MRI, radiomic, inverted papilloma, nasal polyp, olfactory nerve, diagnostic efficacy

## Abstract

**Background and purpose:**

Inverted papilloma (IP) and nasal polyp (NP), as two benign lesions, are difficult to distinguish on MRI imaging and clinically, especially in predicting whether the olfactory nerve is damaged, which is an important aspect of treatment and prognosis. We plan to establish a new biomarker to distinguish IP and NP that may invade the olfactory nerve, and to analyze its diagnostic efficacy.

**Materials and methods:**

A total of 74 cases of IP and 55 cases of NP were collected. A total of 80% of 129 patients were used as the training set (59 IP and 44 NP); the remaining were used as the testing set. As a multimodal study (two MRI sequences and clinical indicators), preoperative MR images including T2-weighted magnetic resonance imaging (T2-WI) and contrast-enhanced T1-weighted magnetic resonance imaging (CE-T1WI) were collected. Radiomic features were extracted from MR images. Then, the least absolute shrinkage and selection operator (LASSO) regression method was used to decrease the high degree of redundancy and irrelevance. Subsequently, the radiomics model is constructed by the rad scoring formula. The area under the curve (AUC), accuracy, sensitivity, specificity, positive predictive value (PPV), and negative predictive value (NPV) of the model have been calculated. Finally, the decision curve analysis (DCA) is used to evaluate the clinical practicability of the model.

**Results:**

There were significant differences in age, nasal bleeding, and hyposmia between the two lesions (*p* < 0.05). In total, 1,906 radiomic features were extracted from T2-WI and CE-T1WI images. After feature selection, using 12 key features to bulid model. AUC, sensitivity, specificity, and accuracy on the testing cohort of the optimal model were, respectively, 0.9121, 0.828, 0.9091, and 0.899. AUC on the testing cohort of the optimal model was 0.9121; in addition, sensitivity, specificity, and accuracy were, respectively, 0.828, 0.9091, and 0.899.

**Conclusion:**

A new biomarker combining multimodal MRI radiomics and clinical indicators can effectively distinguish between IP and NP that may invade the olfactory nerve, which can provide a valuable decision basis for individualized treatment.

## 1. Introduction

Inverted papilloma (IP) is a common benign epithelial-derived tumor of the nasal cavity and sinuses, accounting for approximately 0.5–4.0% of nasal tumors (1); a complete surgical excision is crucial for their efficacy and prognosis (2). Nasal polyp (NP), also known as polypoid degeneration, has a high clinical incidence and can be treated by nasal irrigation or nasal endoscopic surgery combined with glucocorticoid medication (3). Some studies have reported that approximately 60% of patients require multiple intraoperative biopsies before an accurate pathological diagnosis can be made (4). Accurate preoperative diagnosis is critical for patients’ treatment. Symptoms of both lesions can manifest as persistent nasal congestion, runny nose, nasal bleeding, facial pain, and hyposmia, making them clinically difficult to distinguish (5). Olfactory hyposmia is often overlooked in the clinical treatment of IP and NP. IP is characterized by local aggressiveness, high recurrence rate, and malignant transformation, and it easily invades the olfactory nerve through the skull base (2), and NP is often accompanied by chronic rhinosinusitis, histological changes of the mucous membrane secondary to the inflammatory process may reduce the olfactory neurons (6). Once surgically removed, patients’ olfaction is not restored, which can seriously affect their quality of life, so it is beneficial to improve the patient’s prognosis if we intervene in advance for IP and NP that may invade the olfactory nerve.

In recent years, the organic integration of artificial intelligence (AI), computer technology, and medical imaging in the context of big data has led to the rapid development of imaging omics. Radiomics (7) refers to obtaining abundant advanced quantitative imaging features from images, extracting feature data to extend conventional images, and applying suitable machine learning algorithms to construct predictive models by implementing tumor segmentation and feature extraction, and these quantitative features are different from the visual images we perceive, aiding physicians in making rapid diagnoses by providing potential value. In this field, machine learning (ML) algorithms are used to select the best features and develop and improve models, which have the potential to improve predictive power (8). In the last 2 years, studies regarding artificial intelligence in IP have gradually become a hot topic (9–13). In one study, Li et al. (14) designed a deep learning framework through convolutional neural networks to automatically identify IP and NP with high AUC values of 0.95. In another study, Ren et al. (15) used a deep convolutional neural network (CNN) which combines a densely connected convolutional network (DenseNet) and squeeze-and-excitation network (SENet) to classify IP and NP in CT and achieved a relatively high diagnostic value. Although these two study models gain excellent results but did not analyze IP and NP from a clinical perspective. MRI, as one of the common examination methods for sinus tumors, has the advantages of no radiation, high soft tissue resolution, and multiplanar imaging compared with CT, and clearly shows the signal changes of the internal structure of the tumor. IP and NP frequently show a lobulated shape with hyperintensity on T2-WI and isointensity to hypointensity on T1-WI (16). The convoluted cerebriform pattern (CCP) is a reliable MRI feature of IP on CE-T1-WI (17), but not all IPs have such characteristics (18). There are no relevant studies discussing the construction of machine learning models based on multi-parameter MRI to distinguish between the two lesions.

In this study, we aimed to use multimodal MRI sequences of the nasal cavity (T2WI, CE-T1WI) to construct radiomics models combined with clinical indicators, to effectively and highly accurately identify IP and NP that may invade the olfactory nerve, and this helps to provide more comprehensive information for their treatment plans.

## 2. Materials and methods

### 2.1. Patients

This retrospective study included two groups of patients who underwent an MRI examination in the Second Hospital of Jilin University from March 2014 to May 2020 and were confirmed as NP or IP by pathological diagnosis. Ethical approval was obtained, and the informed consent requirement was waived by our institutional reviewing board. Inclusion criteria were as follows: (1) patients who underwent MRI examination and had definite pathological diagnosis result; (2) no history of the nasal cavity or sinus surgery, trauma, or other local treatment; (3) no recurrence or malignant transformation; and (4) complete and clear MRI image. Exclusion criteria were as follows: (1) no pathological diagnosis result; (2) no complete clinical information; and (3) unqualified image: defective, unclear, and abnormal posture.

**Figure 1:**
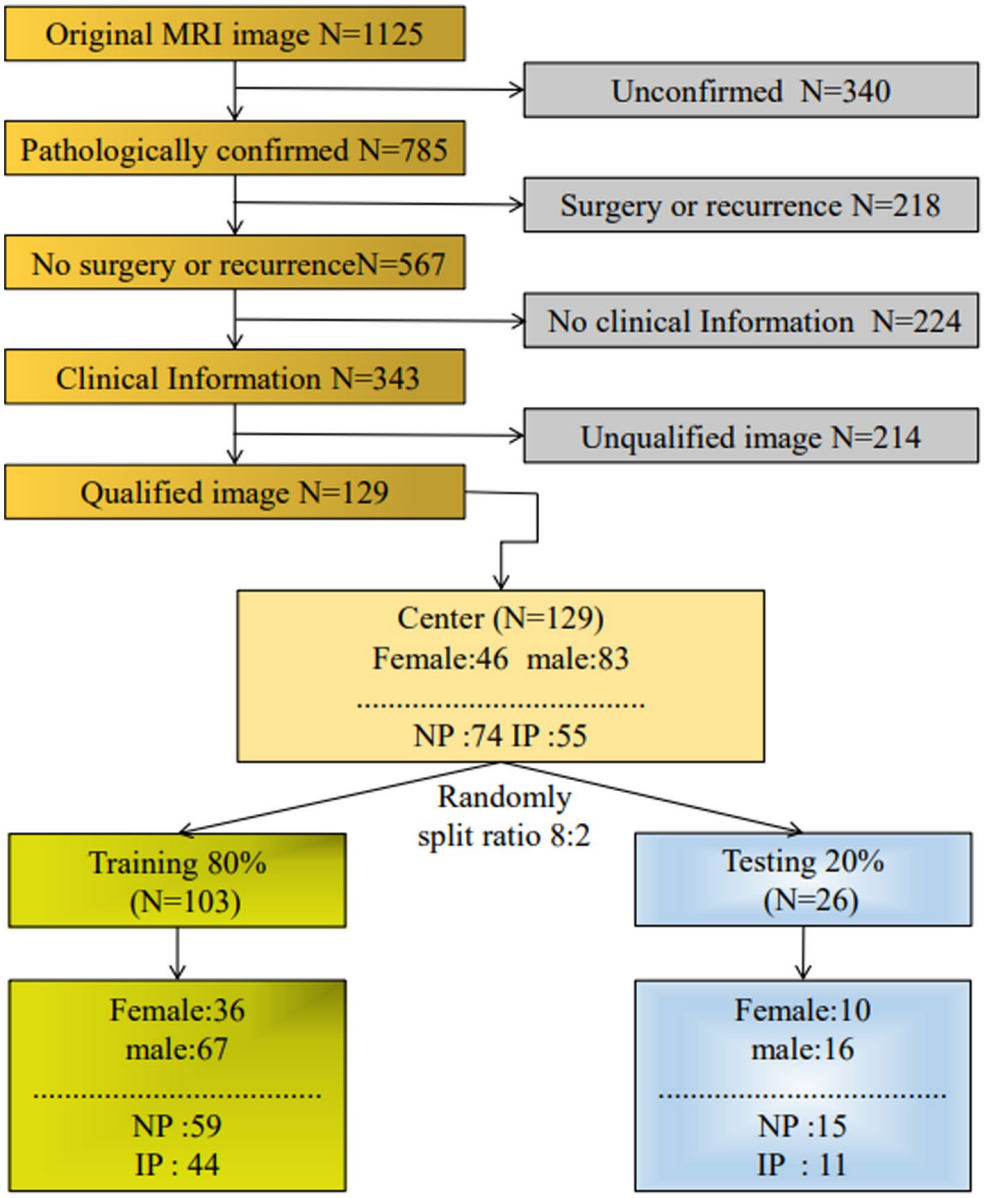
Filter flowchart. Through the inclusion and exclusion criteria of each step, a total of 129 patients were enrolled in the study and randomly assigned to training or testing sets.

As shown in Figure 1, a total of 170 patients’ data were collected from the hospital database, and a total of 129 patients were included in the study. Clinical indicators include persistent nasal congestion, runny nose, nasal bleeding, facial pain, and hyposmia. In addition, individual cases were found to have decreased sense of hearing and tinnitus, considered to be the cause of an oversized involuted papilloma compressing the eustachian tube or inflammatory infiltration, so they are also included. Based on the inclusion criteria, a total of 129 patients, including 74 patients with NP (47 men and 27 women: 43.55 ± 16.81 years; range, 14–85 years) and 55 patients (36 men and 19 women: 52.72 ± 10.44 years; range, 12–82 years) with IP, were randomly assigned to a training or testing cohort to explore and validate the diagnostic performance of the model between NP and IP.

### 2.2. Image acquisition

The equipment for image acquisition was a 3 T MRI system (DiscoveryMR750; GE Healthcare, Waukesha, WI). Axial T2WI (TR/TE 3000–3,500/120-130 ms, NEX 1) images were obtained by conventional plain scan examination. Then, patients were given an injection of 0.1 mmol/kg of contrast-enhancing agent (gadopentetate glucosamine), and axial CE-T1WI images were obtained with an adjusted layer thickness of 4–5 mm, a layer spacing of 0.5 mm, a matrix of 320 × 256, and field of view (FOV) of 20 cm × 20 cm.

### 2.3. Image preprocessing

**Figure 2:**
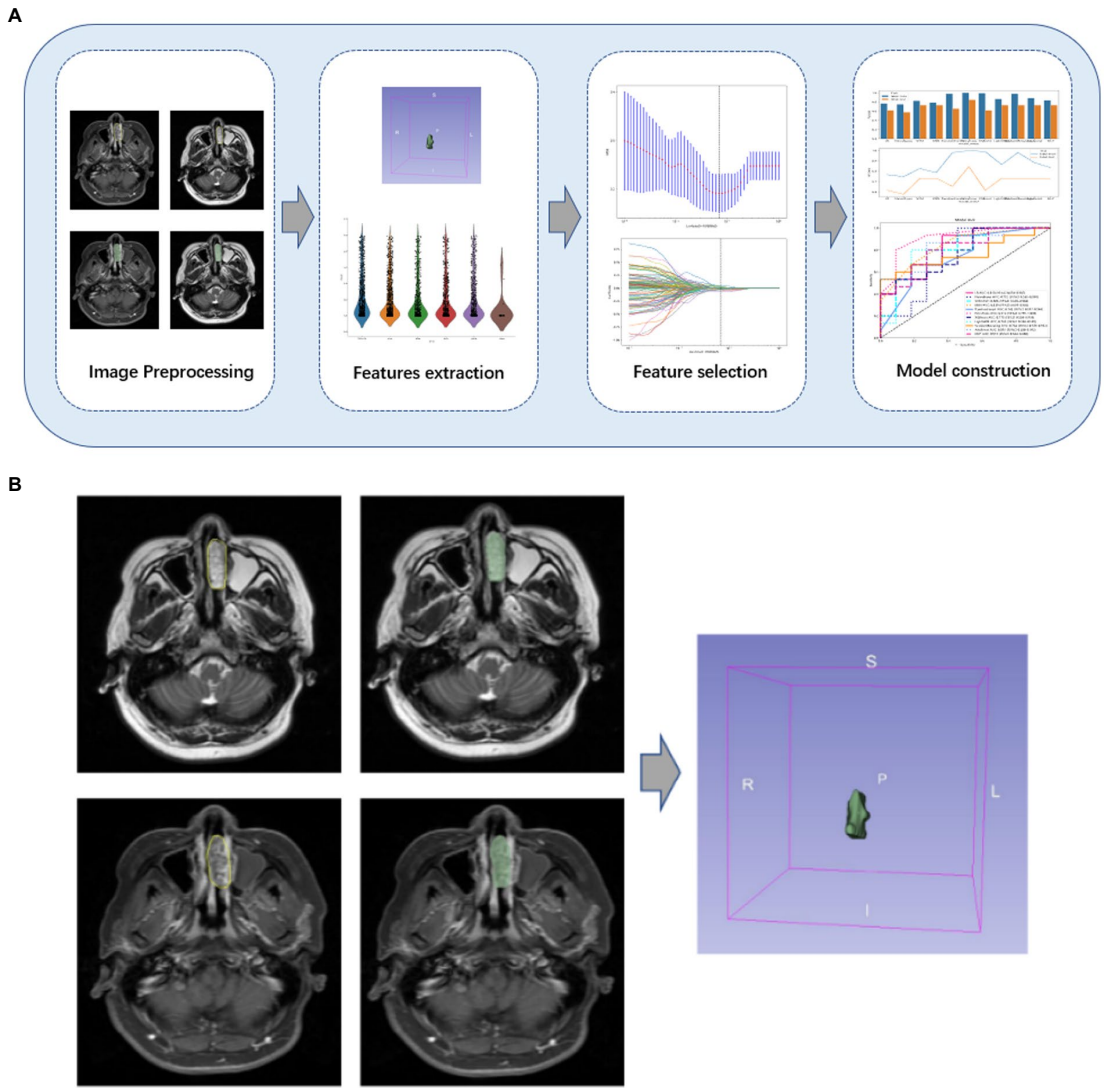
**(A)** Flowchart of the study. An overview of the study workflow including image preprocessing, feature extraction, feature selection, and model construction. **(B)** Some cases of lesions drawn by radiologists. The tumor contour on the CE-T1WI image was determined, and the CE-T1WI image was referred to when sketching the T2-WI image.

The flowchart of image processing, feature extraction, feature selection, and model construction is given in Figure 2A. In this study, open-source medical image processing software (3D Slicer, version 4.11.0)^1^ is used as the analysis platform. First, DICOM data of qualified axial T2-WI and CE-T1WI original scanning images were imported into 3Dslicer software, then the region of interest (ROI) for each slice was drawn, followed by determining the tumor contour on the CE-T1WI image, and finally, the CE-T1WI image was referred when sketching the T2-WI image.

Radiologists with 8 and 20 years of experience, respectively, will work together to assess image quality and confirm the location of the primary tumor. If there is a disagreement, the two radiologists will discuss it and make a decision. The ICC of the radiomic features assessed by different radiologists was calculated. Features with an ICC greater than 0.80 were considered to be in good agreement and reserved for further analysis (19). These two radiologists were blinded to the information about each lesion. Figure 2B shows an example of the manual split process.

### 2.4. Features extraction

**Figure 3:**
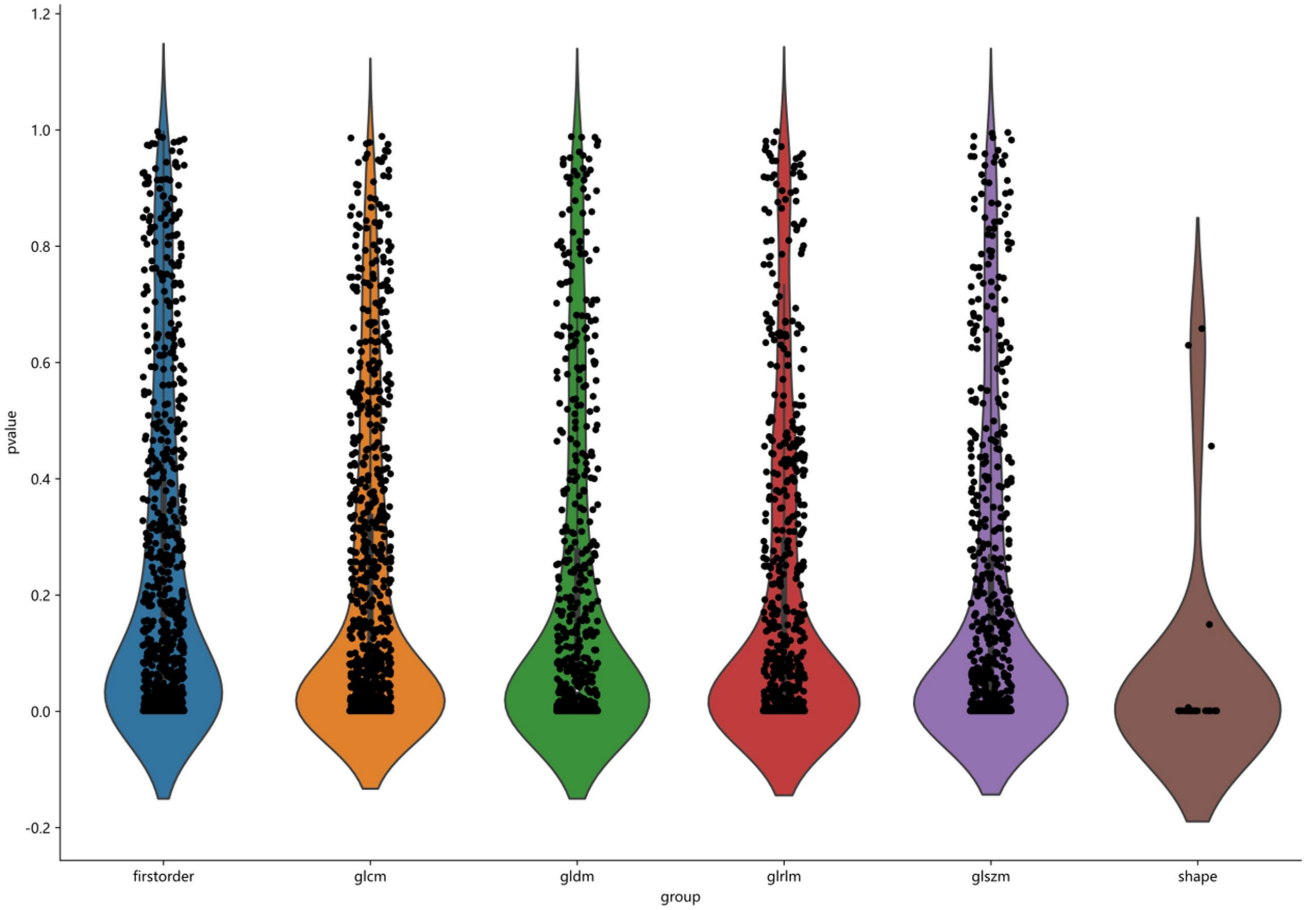
Classification of 1906 features, including first order, glcm, gldm, glrlm, glszm, and shape.

3D Slicer software automatically generates a 3D volume area of interest and saves it in nii or nii.gz format. The features were then extracted using a radiomics module (Pyradiomics) based on Python (version 3.7.10) software. Based on the MRI images, we carried out the pre-processing and image transformation. Many image preprocessing methods were used, including Wavelet, LOG, and Square, and the best parameters are adjusted. After image processing, we obtained a total of 1,906 features from each ROI, as shown in Figure 3, including the three categories as follows: first-order features, shape features, and texture features (20). First-order features (396 features) use basic first-order statistics such as mean, variance, entropy, and standard deviation to describe the pixel intensity and distribution within the ROI. Shape features (14 features) describe the shape and size information of ROI in 2D or 3D, such as volume, diameter, and roundness. Texture features mainly include gray level emphasis, gray level nonuniformity, gray level nonuniformity normalized, gray level variance, and gray level run emphasis, which describe the gray-level relationship between a pixel and surrounding pixels.

### 2.5. Feature selection

A total of 80% of datasets were randomly used as the training set, the remaining were used as the testing set. Before feature selection, all radiomics features were standardized by removing the mean and dividing by its standard deviation with the StandardScaler function. Each set of feature values was converted to a mean of 0 with a variance of 1. The Pearson correlation coefficient (PCC) method is used to reduce the dimensionality of the features and exclude those with a correlation coefficient threshold higher than 0.9. Then, the least absolute shrinkage and selection operator (LASSO) regression method was used to compress the regression coefficients of redundant prediction variables. The best λ, the coefficient of regularization used for the LASSO method, was selected using inner 5-fold cross-validation in the training set *via* minimum average mean square error (MSE). Subsequently, the radiomics parameters with non-zero coefficients in the LASSO model were combined into the *rad-score* formula.

### 2.6. Model construction

Our study explored and verified 11 Ml models, namely, logistic regression (LR), support vector machine (SVM), random forest (RF), AdaBoost, Gradient Boosting (GB), Gaussian Naive Bayesian (NB), K-nearest neighbors (KNN), and ExtraTrees. All data are randomly divided into training and test cohort at a ratio of 8:2. Diagnostic performances of different imaging models were evaluated using the receiver operating characteristic curve. AUC, accuracy, sensitivity, specificity, positive predictive value (PPV), and negative predictive value (NPV) of the model were calculated. The confusion matrix for the test set was constructed based on the predicted values. Finally, decision curve analysis (DCA) is used to evaluate the clinical practicability usefulness of the model.

### 2.7. Statistical analysis

Statistical analyses in this study were conducted in the SPSS software package (version 25.0; IBM, Armonk, NY). Clinical characteristics of all numeric data are statistically described using mean, standard deviation, frequency, and percentage. Two independent sample *t*-tests were used for continuous variables conforming to a normal distribution, the Mann–Whitney U-test was used for skewed distribution, and the ROC analysis was used to evaluate the diagnostic performances of ML classifiers and visual assessment [95% confidence intervals (CIs), specificity, and sensitivity were also calculated]. A *p* < 0.05 was considered statistically significant.

## 3. Results

### 3.1. Clinical characteristics

Patients with NP were significantly younger than those with IP (*p* < 0.05), and patients with IP were more prone to runny nose and hyposmia than those with NP (*p* < 0.05), but there were no significant differences in gender, persistent nasal congestion, runny nose, facial pain, and decreased sense of hearing between the two lesions (*P >* 0.05) (Table 1).

**Table 1:** Characteristics of the patients in all numeric data.

Patient characteristics	NP (*n* = 74)	IP (*n* = 55)	*P*
Age (mean ± SD)	43.55 ± 16.81	52.72 ± 10.44	0.002
Gender			0.820
Male	47	36	
Female	27	19	
Persistent nasal Congestion	67	50	0.943
Runny nose	66	44	0.145
Nasal bleeding	28	31	0.037
Facial pain	31	30	0.155
hyposmia	26	29	0.046
Decreased sense of hearing	8	6	0.986

### 3.2. Reproducibility and feature selection

The ICCs calculated for agreement of features extracted by the two radiologists ranged from 0.865 to 0.968 for T2-WI and from 0.934 to 0.991 for CE-T1W, reflecting good agreement.

**Figure 4:**
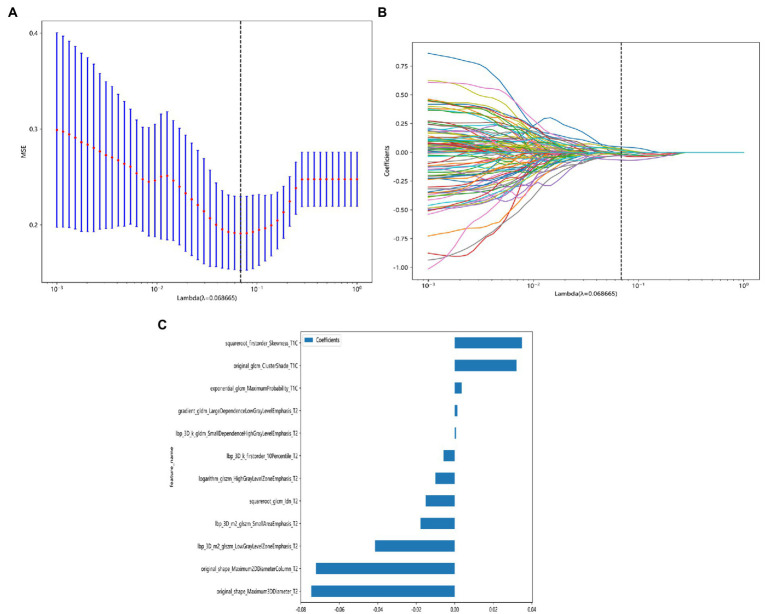
LASSO model dimensionality reduction visualization process. **(A)** Feature dimensionality reduction using LASSO, the horizontal coordinate indicates the value of the penalty coefficient, and the vertical coordinate indicates the change of the binomial deviation size as the value of the coefficient changes. **(B)** Feature dimensionality reduction using LASSO, the lower horizontal coordinate indicates the log(λ) value, the upper horizontal coordinate indicates the number of features corresponding to log(λ), and the vertical coordinate indicates the number of each feature’s weight coefficient. **(C)** Coefficient values of different radiomic signature parameters when constructing rad-score after feature dimensionality reduction using LASSO.

After feature selection with the LASSO method, the radiomic signature label rad-score constructed when the minimum coefficient is taken has the smallest binomial deviation (Figure 4A), when log(λ) = 0.068 (Figure 4B), and the weighting coefficients for constructing the rad-score radiomic features are shown in Figure 4C. The rad-score formula is as follows:

label = 0.5868593143167473 + 0.003605 * exponential_glcm_MaximumProbability_T1C.

+0.032099 * original_glcm_ClusterShade_T1C.

+0.034859 * squareroot_firstorder_Skewness_T1C.

+0.001365 * gradient_gldm_LargeDependenceLowGrayLevelEmphasis_T2.

−0.005804 * lbp_3D_k_firstorder_10Percentile_T2.

+0.000674 * lbp_3D_k_gldm_SmallDependenceHighGrayLevelEmphasis_T2.

−0.041404 * lbp_3D_m2_glszm_LowGrayLevelZoneEmphasis_T2.

−0.017859 * lbp_3D_m2_glszm_SmallAreaEmphasis_T2.

−0.010033 * logarithm_glszm_HighGrayLevelZoneEmphasis_T2.

−0.072139 * original_shape_Maximum2DDiameterColumn_T2.

−0.074584 * original_shape_Maximum3DDiameter_T2.

−0.015069 * squareroot_glcm_Idn_T2.

### 3.3. Diagnostic performance of various classifier models

**Figure 5:**
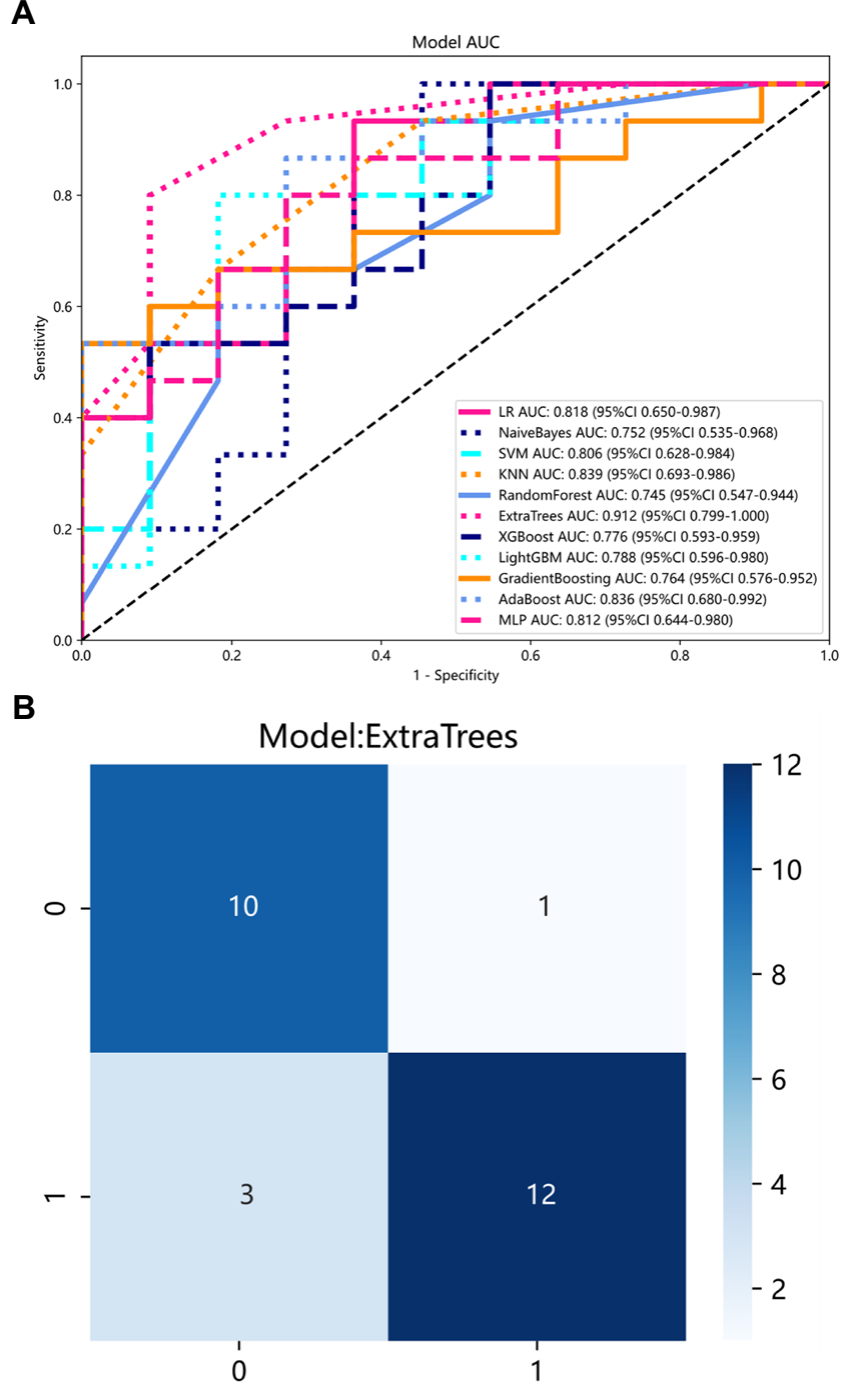
**(A)** ROC curve of 11 models in the test set. Each color represents a model. **(B)** Confusion matrix for ExtraTrees model in the test set.

**Figure 6:**
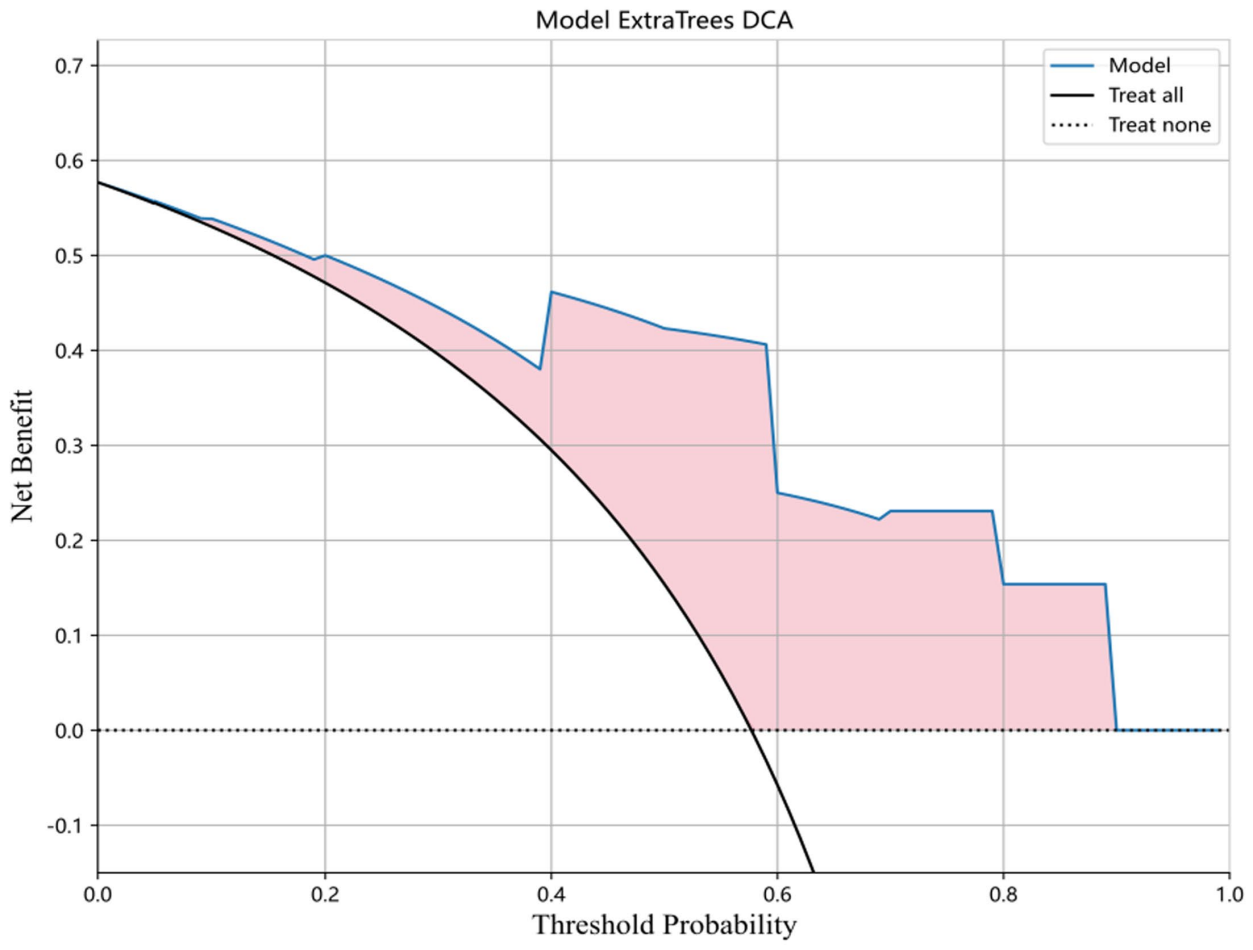
DCA for ExtraTrees model in the test set. The predictive model curves are significantly farther away from the two extreme lines, indicating a good overall net benefit in the population.

The diagnostic performance and cutoff values of various classifier models in discriminating IP from NP in the training and testing sets are summarized in Table 2. As shown in Table 2, the highest AUC (0.9121), accuracy (0.8461), sensitivity (0.8000), and specificity (0.9091) are shown in the ExtraTrees model on the test cohort. The LR, NB, SVM, KNN, RF, XGB, LightGBM, GB, AB, and MLP models also showed excellent AUC performance at 0.8182, 0.7515, 0.8060, 0.8394, 0.7455, 0.7758, 0.7879, 0.7636, 0.8364, and 0.8121, respectively. The performance of the other models in the test cohort was general. AUC and other values were not as good as those of the above seven models. Therefore, in this study, the performance ability of the model was in the following order: ExtraTrees > KNN > AB > LR > MLP > SVM > LightGBM > others. Finally, we summarized the ROC of the 11 types of machine learning models with higher AUC and confusion matrix for the ExtraTrees model in the test set, as shown in Figure 5, as a visual situation analysis table, the confusion matrix indicated that the classification model has a high accuracy rate. As shown in Figure 6, DCA showed that the predictive model curves were significantly farther away from the two extreme lines, indicating a good overall net benefit in the population.

**Table 2:** Average performance of different machine learning models on train cohort (even rows) and test cohort (odd rows).

	model_name	Accuracy	AUC	95% CI	Sensitivity	Specificity	PPV	NPV
0	LR	0.766990	0.873652	0.8071–0.9403	0.898305	0.704545	0.786885	0.738095
1	LR	0.615385	0.818182	0.6498–0.9865	0.933333	0.636364	0.727273	0.533333
2	NaiveBayes	0.747573	0.842450	0.7690–0.9159	0.813559	0.727273	0.836735	0.666667
3	NaiveBayes	0.576923	0.751515	0.5350–0.9681	1.000000	0.545455	0.700000	0.500000
4	SVM	0.825243	0.920647	0.8650–0.9763	0.779661	0.977273	0.805970	0.861111
5	SVM	0.730769	0.806061	0.6277–0.9845	0.800000	0.727273	0.833333	0.642857
6	KNN	0.786408	0.880200	0.8191–0.9413	0.610169	0.954545	0.803279	0.761905
7	KNN	0.730769	0.839394	0.6927–0.9861	0.666667	0.818182	0.833333	0.642857
8	RandomForest	0.980583	1.000000	nan - nan	1.000000	1.000000	0.967213	1.000000
9	RandomForest	0.653846	0.745455	0.5466–0.9443	0.666667	0.818182	0.714286	0.583333
10	ExtraTrees	1.000000	1.000000	nan - nan	1.000000	1.000000	1.000000	1.000000
11	ExtraTrees	0.846154	0.912121	0.7992–1.0000	0.800000	0.909091	0.823529	0.888889
12	XGBoost	0.990291	1.000000	nan - nan	1.000000	1.000000	0.983333	1.000000
13	XGBoost	0.615385	0.775758	0.5927–0.9588	1.000000	0.454545	0.692308	0.538461
14	LightGBM	0.864078	0.919106	0.8655–0.9727	0.932203	0.795455	0.846154	0.894737
15	LightGBM	0.730769	0.787879	0.5961–0.9797	0.800000	0.818182	0.750000	0.700000
16	GradientBoosting	0.980583	0.993066	0.9792–1.0000	1.000000	0.977273	0.983051	0.977273
17	GradientBoosting	0.730769	0.763636	0.5757–0.9516	0.533333	1.000000	0.833333	0.642857
18	AdaBoost	0.883495	0.966294	0.9370–0.9956	0.881356	0.977273	0.873016	0.900000
19	AdaBoost	0.730769	0.836364	0.6802–0.9925	0.866667	0.727273	0.785714	0.666667
20	MLP	0.834951	0.896379	0.8368–0.9560	0.813559	0.840909	0.838710	0.829268
21	MLP	0.730769	0.812121	0.6438–0.9804	0.800000	0.727273	0.785714	0.666667

## 4. Discussion

Inverted papilloma (IP) and NP have a high probability of recurrence, therefore, efficacious preoperative assessment of these lesions is crucial for symptomatic treatment and to reduce the recurrence rate (16). Pathological biopsy is the gold standard, but it is an invasive and limited examination. MRI has a very high soft tissue resolution and can clearly distinguish between the tumor itself and the surrounding environment, which can compensate for the limitations of tissue biopsy (21). Even though traditional radiology diagnostic methods are convenient and cost-effective in routine clinical practice (13), in a large number of cases, the clinical radiological characteristics alone are not enough to accurately distinguish IP and NP.

The analysis of clinical characteristics shows that IP is more common in the elderly and the incidence of nasal bleeding is greater than that of NP, which is consistent with the results of this study. Interestingly, hyposmia is statistically significant between the two, which has not been mentioned in previous studies. Both IP and NP can occur due to conductive olfactory disturbance caused by nasal tract obstruction, which is manifested by a decrease in olfactory hormone molecules reaching the olfactory nerve epithelium, while IP can also occur due to the neurological olfactory disturbance caused by damage to the olfactory nerve or olfactory center, which is manifested by an oversized tumor squeezing the olfactory bundle or the mucosal receptors at the top of the nasal cavity, the upper nasal septum, and the medial aspect of the superior turbinate innervated by the olfactory nerve (22).

Although olfaction in humans is less appreciated than other senses, it affects our lives all the time (23). Because the olfactory nerve is the cranial nerve exposed to the external environment, it is vulnerable to tumor compression and inflammatory stimulation, resulting in reduced olfactory function (24). Olfactory nerve injury cannot be recovered if there is a prolonged attack of the lesion and the degree of damage is relatively severe (25). Most nasal cavity or sinus tumors may damage the olfactory nerve, and in case of olfactory disturbance, timely treatment is required. Our study also aims to highlight the necessity of predicting olfactory nerve invasion in clinical practice.

Radiomics is automated and objective, and thus does not rely on human-derived measurements of image features (26). In our study, the best 12 radiomic features that could distinguish IP from NP included eight texture features, two shape features, and two first-order features. Among them, the texture features, which reflect gray-level nonuniformity account for a large proportion, may be explained by a higher heterogeneity of the images (27). This may be due to differences in tissue composition. In the case of IP, tumors derived from the Schneiderian membrane grow and replace areas of the mucous, serous glands, and ducts (28), whereas, in the case of nasal polyps, it contains mainly fibrin and water (29). A small number of shape features is related to an irregular multinodular mass observed under the endoscopy (30). Radiological features provide more systematic, comprehensive, and quantitative information on tumor heterogeneity than traditional morphological features, which can help explain the potential relationship between pathophysiological and radiology imaging phenotypes (31).

We build a radiomics model based on preoperative multimodality MRI imaging parameters to construct radiomic signature labels through feature downscaling and multiple model optimization. Among all models, ExtraTrees got the most satisfactory result of 0.9121 in AUC. In Li’s research, by using a neural network and analyzing its ability to discriminate the differences, the diagnosis between IP and NP reached a sensitivity of 90.60%, a specificity of 86.40%, and an AUC of 0.884, which are similar to our diagnostic efficacy. In another study of automatic identification of IP and NP by convolutional neural networks, the result reached an accuracy of 89.30%, a sensitivity of 89.01%, a specificity of 89.70%, and an AUC of 0.95. Numerically, the results were slightly higher than the diagnostic performance of our study. It may be due to differences in radiomics models or algorithms, but it basically reaches the diagnostic performance of diagnostic radiologists. We combined clinical indicators through a multimodal study, making the data more comprehensive, providing more useful information for the clinic, controlling for bias due to missing clinical information, and more convincing. Compliance with the principle of early diagnosis and early treatment can clarify the disease as early as possible and achieve an improved prognosis.

This study also has some limitations. On the one hand, it is a retrospective study in a single-center, small data set, and a more rigorous study using multicenter and large-scale data sets is needed to avoid overfitting. On the other hand, we only analyzed the radiomics of T2WI and CE-TIWI sequences, and in future, we will integrate clinical data and radiomic features of other MRI sequences, such as TIWI and diffusion-weighted imaging (DWI), to further improve the diagnostic accuracy of the model.

## 5. Conclusion

In summary, a new biomarker combining multimodal MRI radiomics and clinical indicators can effectively distinguish between IP and NP that may invade the olfactory nerve and can be a valuable addition to routine clinical practice, thus providing a more accurate and objective basis for individualized treatment decisions, showing the potential application value and prospects of radiomic models in nasal cavity diseases.

## Data availability statement

The raw data supporting the conclusions of this article will be made available by the authors, without undue reservation.

## Ethics statement

The studies involving human participants were reviewed and approved by the ethics committee of the Second Hospital of Jilin University (no. SB-2021-012). Written informed consent for participation was not required for this study in accordance with the national legislation and the institutional requirements.

## Author contributions

QY: conceived and designed the study. LD and QH: acquisition of data and drafting the manuscript. LD, QY, and QH: analysis and interpretation of data. All authors contributed to the article and approved the submitted version.

## Funding

Department of Science and Technology of Jilin Province (20200404200YY).

## Conflict of interest

The authors declare that the research was conducted in the absence of any commercial or financial relationships that could be construed as a potential conflict of interest.

## Publisher’s note

All claims expressed in this article are solely those of the authors and do not necessarily represent those of their affiliated organizations, or those of the publisher, the editors and the reviewers. Any product that may be evaluated in this article, or claim that may be made by its manufacturer, is not guaranteed or endorsed by the publisher.

## Footnotes

^1^ http://www.slicer.org

## References

[1] Melroy CT Senior BA. Benign sinonasal neoplasms: a focus on inverting papilloma. Otolaryngol Clin N Am. (2006) 39:601. doi: 10.1016/j.otc.2006.01.005 16757234

[2] Robin TP Jones BL Gordon OM Phan A Abbott D McDermott JD. A comprehensive comparative analysis of treatment modalities for Sinonasal malignancies. Cancer. (2017) 123:3040–9. doi: 10.1002/cncr.30686, PMID: 28369832 PMC6234843

[3] Fokkens WJ Lund VJ Mullol J Bachert C Alobid I Baroody F. European position paper on rhinosinusitis and nasal polyps 2012. Rhinology. (2012) 50:1–12. doi: 10.4193/Rhino12.000, PMID: 22469599

[4] Blandamura S Marioni G de Filippis C Giacomelli L Segato P Staffieri A. Temporal bone and sinonasal inverted papilloma - the same pathological entity? Arch Otolaryngol Head Neck Surg. (2003) 129:553–6. doi: 10.1001/archotol.129.5.553, PMID: 12759269

[5] Lisan Q Laccourreye O Bonfils P. Sinonasal inverted papilloma: From diagnosis to treatment. Eur Ann Otorhinolaryngol Head Neck Dis. (2016) 133:337–41. doi: 10.1016/j.anorl.2016.03.006 27053431

[6] Ottaiano AC Freddi TAL Lucio LL. The olfactory nerve: anatomy and pathology. Semin Ultrasound CT MRI. (2022) 43:371–7. doi: 10.1053/j.sult.2022.04.001, PMID: 36116849

[7] Lambin P Rios-Velazquez E Leijenaar R Carvalho S van Stiphout RGPM Granton P. Radiomics: extracting more information from medical images using advanced feature analysis. Eur J Cancer. (2012) 48:441–6. doi: 10.1016/j.ejca.2011.11.036, PMID: 22257792 PMC4533986

[8] Han Q Du L Mo Y Huang C Yuan Q. Machine learning based non-enhanced CT radiomics for the identification of orbital cavernous venous malformations: an innovative tool. J Craniofac Surg. (2022) 33:814–20. doi: 10.1097/scs.0000000000008446, PMID: 35025826

[9] Suh CH Lee JH Chung MS Xu XQ Sung YS Chung SR. MRI predictors of malignant transformation in patients with inverted papilloma: a decision tree analysis using conventional imaging features and histogram analysis of apparent diffusion coefficients. Korean J Radiol. (2021) 22:751–8. doi: 10.3348/kjr.2020.0576, PMID: 33289362 PMC8076834

[10] Zhang D Zhang J Zhou J Xu J Guo Y Zhang Z. Predictive value of magnetic resonance imaging multi-parametric analysis for malignant transformation of Sinonasal inverted papilloma: a comprehensive prediction model. Curr. Med. Imaging. (2022) 19:596–604. doi: 10.2174/1573405618666220928091936 36173080

[11] Li Z Xian M Guo J Wang CS Zhang L Xian J. Dynamic contrast-enhanced MRI can quantitatively identify malignant transformation of sinonasal inverted papilloma. Br J Radiol. (2022) 95:20211374. doi: 10.1259/bjr.20211374 35234501 PMC10996421

[12] Yan Y Liu Y Tao J Li Z Qu X Guo J. Preoperative prediction of malignant transformation of Sinonasal inverted papilloma using MR Radiomics. Front. Oncol. (2022) 12:870544. doi: 10.3389/fonc.2022.870544, PMID: 35402254 PMC8983836

[13] Zhang Z Yu L Jiang J Wang L Zhou S Hao D. Development and validation of a clinical prediction model to diagnose Sinonasal inverted papilloma based on computed tomography features and clinical characteristics. Ent Ear Nose Throat J. (2022):014556132211344. doi: 10.1177/01455613221134421, PMID: 36264012

[14] Li X Zhao H Ren T Tian Y Yan A Li W. Inverted papilloma and nasal polyp classification using a deep convolutional network integrated with an attention mechanism. Comput Biol Med. (2022) 149:105976. doi: 10.1016/j.compbiomed.2022.105976, PMID: 36067631

[15] Ren T Li X Tian Y Li W. Deep learning framework for preoperative recognition of inverted papilloma and nasal polyp. Ieee Access. (2021) 9:120502–11. doi: 10.1109/access.2021.3099687

[16] Tatekawa H Shimono T Ohsawa M Doishita S Sakamoto S Miki Y. Imaging features of benign mass lesions in the nasal cavity and paranasal sinuses according to the 2017 WHO classification. Jpn J Radiol. (2018) 36:361–81. doi: 10.1007/s11604-018-0739-y, PMID: 29696477

[17] Jeon TY Kim HJ Chung SK Dhong HJ Kim HY Yim YJ. Sinonasal inverted papilloma: value of convoluted cerebriform pattern on MR imaging. Am J Neuroradiol. (2008) 29:1556–60. doi: 10.3174/ajnr.A1128, PMID: 18499786 PMC8119066

[18] Eid M Eissa L. Imaging of sino-nasal inverted papilloma: how can we emphasize the usefulness of the "striated pattern" sign? Egypt J Radiol Nucl Med. (2020) 51:29. doi: 10.1186/s43055-020-0134-4

[19] Limkin EJ Sun R Dercle L Zacharaki EI Robert C Reuze S. Promises and challenges for the implementation of computational medical imaging (radiomics) in oncology. Ann Oncol. (2017) 28:1191–206. doi: 10.1093/annonc/mdx034, PMID: 28168275

[20] Peng HC Long FH Ding C. Feature selection based on mutual information: criteria of max-dependency, max-relevance, and min-redundancy. IEEE Trans Pattern Anal Mach Intell. (2005) 27:1226–38. doi: 10.1109/tpami.2005.159, PMID: 16119262

[21] Gillies RJ Kinahan PE Hricak H. Radiomics: images are more than pictures, they are data. Radiology. (2016) 278:563–77. doi: 10.1148/radiol.2015151169, PMID: 26579733 PMC4734157

[22] Upadhya IB Rao K. Sinonasal inverted papilloma: a narrative review. Indian J Otolaryngol Head Neck Surg. (2022) 74:1017–22. doi: 10.1007/s12070-020-02089-0, PMID: 36452822 PMC9701979

[23] Fornazieri MA Pinto Borges BB Pinto Bezerra TF Pinna FR Voegels RL. Main causes and diagnostic evaluation in patients with primary complaint of olfactory disturbances. Braz J Otorhinolaryngol. (2014) 80:202–7. doi: 10.1016/j.bjorl.2014.02.001, PMID: 25153103 PMC9535487

[24] Hu B Zhang JY Gong MD Deng YQ Cao YJ Xiang YZ. Research Progress of olfactory nerve regeneration mechanism and olfactory training. Ther Clin Risk Manag. (2022) 18:185–95. doi: 10.2147/tcrm.S354695, PMID: 35281777 PMC8906848

[25] Abolmaali N Gudziol V Hummel T. Pathology of the olfactory nerve. Neuroimaging Clin N Am. (2008) 18:233. doi: 10.1016/j.nic.2007.10.002 18466830

[26] Akkus Z Galimzianova A Hoogi A Rubin DL Erickson BJ. Deep learning for brain MRI segmentation: state of the art and future directions. J Digit Imaging. (2017) 30:449–59. doi: 10.1007/s10278-017-9983-4, PMID: 28577131 PMC5537095

[27] Xie T Chen X Fang J Kang H Xue W Tong H. Textural features of dynamic contrast-enhanced MRI derived model-free and model-based parameter maps in glioma grading. J Magn Reson Imaging. (2018) 47:1099–111. doi: 10.1002/jmri.25835, PMID: 28845594

[28] Wood JW Casiano RR. Inverted papillomas and benign nonneoplastic lesions of the nasal cavity. Am J Rhinol Allergy. (2012) 26:157–63. doi: 10.2500/ajra.2012.26.3732, PMID: 22487294 PMC3906506

[29] London NR Jr Reh DD. Differential diagnosis of chronic rhinosinusitis with nasal polyps. Adv Otorhinolaryngol. (2016) 79:1–12. doi: 10.1159/000444957 27466841

[30] Cho HS Kim KS. Nasal obstruction due to septochoanal polyp. Braz J Otorhinolaryngol. (2014) 80:362–3. doi: 10.1016/j.bjorl.2014.05.024, PMID: 25183189 PMC9444637

[31] Trivizakis E Manikis GC Nikiforaki K Drevelegas K Constantinides M Drevelegas A. Extending 2-D convolutional neural networks to 3-D for advancing deep learning cancer classification with application to MRI liver tumor differentiation. IEEE J Biomed Health Inform. (2019) 23:923–30. doi: 10.1109/jbhi.2018.2886276, PMID: 30561355

